# Association Between Hemoglobin Levels and Hemorrhagic Stroke Severity and In‐Hospital Outcomes: A Multi‐Centre Cross‐Sectional Study on Recovery and Prognosis in Pakistan

**DOI:** 10.1002/brb3.71715

**Published:** 2026-09-15

**Authors:** Ayesha Johar, Ayesha Ahmad, Javeria Akhter, Wajiha Aftab, Aneika Ali, Maryam Asghar Jamal, Maham Jawad, Rida Noor, Hafsa Majeed, Adnan Khanm, Kamil Ahmad Kamil

**Affiliations:** ^1^ Khyber Medical University Peshawar Pakistan; ^2^ Indus Hospital and Health Network Karachi Pakistan; ^3^ Dow International Medical College Dow University of Health Sciences Karachi Pakistan; ^4^ Gomal Medical College Dera Ismail Khan KPK Pakistan; ^5^ Khyber Girls Medical College Peshawar Pakistan; ^6^ Fatima Memorial Hospital and College of Medicine & Dentistry Lahore Pakistan; ^7^ Bolan Medical College Quetta Pakistan; ^8^ King Edward Medical University Lahore Pakistan; ^9^ Chief, Department of Internal Medicine Nasrat Bashir Curative Hospital Kandahar Afghanistan

## Abstract

**Objective:**

To evaluate the association between admission hemoglobin levels and in‐hospital functional outcomes in patients with acute hemorrhagic stroke in Pakistan.

**Background:**

Abnormal hemoglobin levels have an impact on cerebral perfusion and oxygen delivery to the brain, potentially affecting the recovery in stroke patients. Both anemia and polycythemia can impact neurological outcomes, yet evidence is not yet conclusive. This study examined the relationship between Hb levels, hemorrhagic stroke severity, and clinical outcomes.

**Design/Methods:**

A retrospective, multi‐center cross‐sectional study was conducted among adults admitted with acute hemorrhagic stroke. Hemoglobin levels measured within 24 h of admission were divided into three groups that are low (<12 g/dL in females, <13 g/dL in males), normal, and high. Patients with high hemoglobin [*n* = 1] were excluded due to insufficient sample size. The sample size was calculated using the standard formula for cross‐sectional studies at a 95% confidence interval and 5% margin of error. The minimum required sample size was 384 participants; however, 400–450 participants were included to compensate for non‐response and incomplete data. Stroke severity was assessed using the NIH Stroke Scale (NIHSS) and functional outcome by the Modified Rankin Scale (mRS) at the time of discharge. Baseline characteristics were summarized descriptively. Group comparisons used Pearson *χ* ^2^ with Cramér's *V* for categorical variables and Mann–Whitney *U* or Kruskal–Wallis tests for continuous or ordinal data. Multivariable logistic regression identified independent predictors of favorable outcome (mRS 0–2) and mortality, adjusting for age, sex, NIHSS, and comorbidities. Model calibration was evaluated using the Hosmer–Lemeshow test and discrimination with Nagelkerke *R* ^2^. Non‐linearity was assessed using restricted cubic splines. NIHSS was retained as a core adjustment variable in all models.

**Results:**

We included 445 stroke patients at discharge only (Hb‐low 330 [74.2%], Hb‐normal 114 [25.6%], and Hb‐high 1). Baseline severity and outcomes were similar by Hb group (NIHSS 9.74 ± 7.02 vs. 10.64 ± 6.25; mRS 2.15 ± 2.20 vs. 2.25 ± 1.78), with deaths 32/330 (9.7%) versus 4/114 (3.5%). Later hospital arrival was associated with higher mRS (χ^2^ p < 0.0001) and mortality (χ^2^ p = 0.013). Diabetes (p < 0.001), hypertension (p = 0.0001), and older age (p = 0.0049) were linked to poorer outcomes, whereas Hb category showed no difference in mRS (p = 0.257) or NIHSS (p = 0.195). In multivariable models, Hb (low vs. normal) was not independently associated with favorable outcomes (aOR 0.77, 95% CI 0.43–1.38, p = 0.378) or mortality (aOR 2.15, 95% CI 0.78–5.91, p = 0.139). NIHSS remained the dominant predictor (favorable outcome: aOR 0.77 per point, p < 0.001; mortality: aOR 1.07 per point, p = 0.019), with diabetes also increasing death risk (aOR 2.58, p = 0.014). No evidence of non‐linearity was found [p = 0.41].

**Conclusions:**

Admission hemoglobin levels were not independently associated with in‐hospital functional outcome or mortality in this cohort. Stroke severity and diabetes were the strongest predictors of poor prognosis. Routine hemoglobin assessment remains clinically relevant, but targeted correction of hemoglobin may not directly improve outcomes in patients. Findings are limited to the in‐hospital period and should not be extrapolated to long‐term recovery.

## Graphical abstract

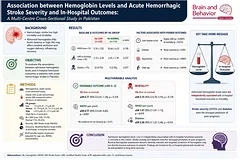

## 1 Introduction

Stroke continues to pose a considerable global health challenge, claiming millions of lives each year and leaving countless people with long‐term disabilities that significantly impair quality of life and functional independence (WHO EMRO 2025). Hemorrhagic stroke accounts for 20% of all stroke cases, with intracerebral hemorrhage being the most common subtype. Despite its lower incidence compared to ischemic stroke, it carries a disproportionately higher burden of mortality, neurological deterioration, and long‐term disability (Montaño et al. 2021; Krishnamurthi et al. 2013). Acute spontaneous intracerebral hemorrhage often results in severe neurologic deficits due to hematoma expansion, cerebral edema, and increased intracranial pressure. The overall prognosis for these patients largely depends on critical factors such as hemorrhagic size, location, comorbidities, and timely interventions like blood pressure control and coagulation reversal (Cordonnier et al. 2018). Moreover, the stroke burden is considerably high in low‐ and middle‐income countries. It is largely due to limited access to acute care facilities, lower public awareness regarding stroke symptoms and prevention, and increasing prevalence of vascular risk factors such as diabetes and hypertension (Feigin et al. 2021). Given its devastating impact and complex pathophysiology, a deeper understanding of prognostic determinants in hemorrhagic stroke is essential to improve clinical outcomes and guide effective management strategies.

Hemoglobin plays an essential role in delivering sufficient oxygen to the brain, and disruption in its balance is increasingly acknowledged as a critical determinant of stroke outcomes. Specifically, low hemoglobin levels have been demonstrated to adversely affect stroke prognosis by diminishing blood oxygen transport capacity and compromising cerebral blood flow. These changes intensify brain tissue hypoxia and aggravate secondary neuronal injury (Barlas et al. 2016). This hypoxic state initiates a series of harmful physiological responses, such as increased oxidative stress resulting from mitochondrial impairment and the generation of reactive oxygen species. Additionally, it leads to endothelial dysfunction, which compromises the integrity of the blood‐brain barrier, and triggers the activation of inflammatory cascades. These interconnected mechanisms collectively exacerbate neuronal injury and worsen neurological outcomes after stroke. (Obeagu and Obeagu 2025). However, high hemoglobin levels are also associated with poor outcomes in hemorrhagic stroke. Elevated hemoglobin levels may worsen outcomes through heme‐mediated oxidative stress, platelet dysfunction, and iron‐related coagulopathy, contributing to larger hematoma volume and poorer functional recovery (Zhang et al. 2022). Despite the growing evidence, the association between hemoglobin levels and stroke outcome remains inconclusive, with findings varying across population, stroke subtypes, and methodological approaches. Barlas et al. have demonstrated that anemia is independently associated with increased mortality following stroke, even after adjustment for potential confounding factors. Furthermore, elevated hemoglobin levels have also been associated with adverse clinical outcomes, supporting the possibility of a U‐shaped relationship between hemoglobin concentration and stroke mortality (2016). As a result, the association between hemoglobin levels and functional outcomes in hemorrhagic stroke remains uncertain; therefore, further studies are required to clarify its role in patient prognosis and clinical recovery.

In Pakistan, stroke represents a major public health concern, with an estimated incidence of approximately 250 cases per 100,000 people annually (Farooq et al. 2021). Hemorrhagic stroke accounts for a considerable proportion of these cases and is associated with mortality rates approaching 21.7%. This high mortality is largely driven by the high prevalence of hypertension and diabetes in the population (Khan et al. 2016). Concurrently, anemia remains highly prevalent in Pakistan, particularly among younger women, pregnant women, and those from lower socioeconomic backgrounds, thereby further complicating the country's health landscape (Ashraf et al. 2024). Despite the coexistence of these clinically significant factors, there is a notable scarcity of local evidence examining the relationship between hemoglobin levels and outcomes in hemorrhagic stroke patients in Pakistan. Specifically, limited research has explored how variations in hemoglobin concentrations at the time of admission may influence the initial severity of hemorrhagic stroke, as well as subsequent in‐hospital outcomes such as mortality, length of hospital stay, and functional disability at discharge. Understanding this potential relationship may be particularly relevant in resource‐limited settings like Pakistan, where inexpensive, widely available, and routinely measured laboratory parameters such as hemoglobin levels could serve as valuable tools for early risk stratifications. Such parameters could assist clinicians in identifying high‐risk patients promptly and guiding clinical decision‐making, ultimately improving stroke management and outcomes even in the absence of advanced diagnostic techniques.

Therefore, this multicenter cross‐sectional study was designed to evaluate the association between admission hemoglobin levels and hemorrhagic stroke severity, functional recovery, and in‐hospital outcomes among patients admitted with acute hemorrhagic stroke in Pakistan. The study further sought to determine whether abnormally low or high hemoglobin levels independently predicted prognosis after adjustments for established clinical risk factors and stroke severity measures.

## 2 Materials and Methods

A retrospective, multi‐center hospital‐based cross‐sectional analytical study was conducted to evaluate the association between hemoglobin levels and in‐hospital stroke outcomes in adult patients. The study was carried out in the medical and stroke units of tertiary care teaching hospitals across multiple provinces in Pakistan, which provide acute management and follow‐up care for stroke patients. Our Independent Variable was Hemoglobin. Hemoglobin was categorized using WHO sex‐specific cutoffs. The Normal Hb level was 13 to 17 g/dl, with 13 considered as the lowest and 17 as the highest values for males and 12 to 15 g/dl, with <12 as the lowest values and >15 as the highest values for females; otherwise, normal. Admission hemoglobin within 24 h was selected because it reflects early physiological status during acute hemorrhagic stroke presentation. Serial measurements were not collected, and we could not assess hemoglobin trajectories. Patients with high Hb [*n* = 1] were excluded due to insufficient sample size.

The Dependent variables were: Stroke severity (NIHSS score), functional outcome (mRS score), and in‐hospital mortality. The mRS score of 0–2 at discharge was considered a good outcome, and score of 3–6 or death was considered poor. The 90‐day mRS was not routinely available across centers, so conclusions are limited to the in‐hospital period. NIHSS is a validated predictor of mortality and disability in acute stroke and was retained as a core adjustment variable in all regression models (Adams et al. 1999; Lyden 2017). Covariates included age, sex, NIHSS, GCS, IVH, pre‐stroke mRS, transfusion history, and comorbidities. Important radiological prognostic variables, including hematoma volume, hemorrhage location, intraventricular extension, and perihematomal edema, were unavailable across all participating centers and therefore could not be incorporated into multivariable models. As this was a cross‐sectional study, temporal relationships between hemoglobin levels and outcomes could not be established.

### 2.1 Sample Size Justification

Because no prior estimates were available for the association between admission hemoglobin levels and hemorrhagic stroke outcomes specifically in the Pakistani population, a formal prior sample size calculation for hypothesis testing (e.g., comparing low vs. normal hemoglobin) could not be performed. Instead, the sample size was determined pragmatically based on the expected number of eligible patients during the study period across participating centers. We aimed to enroll a minimum of 384 participants, which is the standard requirement for a cross‐sectional study estimating a proportion with a 95% confidence interval and a 5% margin of error (using p = 0.5). This target was exceeded, with 445 consecutive eligible patients enrolled.

The final distribution (330 low Hb, 114 normal Hb, 1 high Hb) allowed for multivariable adjustment but had limited power to detect small effect sizes. Specifically, the observed mortality rates (9.7% in low Hb vs. 3.5% in normal Hb) correspond to an unadjusted odds ratio of approximately 3.0. The study had sufficient power to detect such a large effect, but the adjusted odds ratio of 2.15 (95% CI 0.78–5.91) was not statistically significant, partly due to the wide confidence interval reflecting limited precision. For smaller but clinically meaningful effects (e.g., OR 1.5–2.0), the study was underpowered. No post‐hoc power calculation was performed, as post‐hoc power is mathematically determined by the observed p‐value and effect size and is not considered informative by contemporary statistical guidelines (Hoenig and Heisey 2001). The single patient with high hemoglobin was excluded from inferential analyses because the sample size was insufficient for meaningful comparison.

Therefore, while the null findings for hemoglobin should be interpreted with caution regarding potential Type II error, the study was adequately powered to detect only moderate‐to‐large associations. This limitation is acknowledged in the Discussion.

### 2.2 Inclusion and Exclusion Criteria

Inclusion Criteria:

- Patients aged ≥18 years.
- Confirmed hemorrhagic stroke diagnosis via clinical evaluation and imaging.
- Hemoglobin levels within 24 h of presentation.

Exclusion Criteria:

- Patients with known hematological disorders.
- Patients who received blood transfusions or had active bleeding within one month before stroke onset.
- Patients in the high hemoglobin category [*n* = 1] were excluded from inferential analyses due to insufficient sample size.
- Recent transfusions, hematologic disorders, and active bleeding were excluded because they could artificially alter hemoglobin independent of stroke physiology.

Ethical approval was obtained from the institutional review board (IRB) of different medical colleges and their tertiary teaching hospitals throughout different provinces of Pakistan. Informed consent was obtained from all participants or their legal guardians. Data was stored securely and used solely for research purposes. Participants could opt out from the study at any time without any effect on their medical care. Data were derived from hospital records, patient interviews and subsequent assessments. We used a structured questionnaire to obtain the information, which included demographic information such as age, sex, and other health problems, the NIHSS score to measure how bad the stroke was; hemoglobin levels to put them in the low, normal. or high range; and the Modified Rankin Scale (mRS) to measure how well they were doing at discharge.

### 2.3 Data Analysis

All analyses were performed using R (version 4.5.1; R Foundation for Statistical Computing, Vienna). Prior to analysis, data cleaning, checking for inconsistencies, and initial coding were performed in Microsoft Excel. Missing data were minimal and handled using a complete‐case (listwise deletion) approach, with no imputation performed. Patients were stratified into hemoglobin (Hb) categories (low and normal), with the Hb‐high group (*n* = 1) excluded from comparative analyses due to insufficient sample size.

Continuous variables were assessed for normality and summarized as mean ± standard deviation (SD) or median with interquartile range (IQR), as appropriate, while categorical variables were presented as frequencies and percentages. Baseline characteristics, clinical presentation, complications, and outcomes were compared between Hb groups using Pearson's chi‐square test for categorical variables, with effect sizes reported using Cramér's *V*, and the Mann–Whitney *U* test for continuous variables such as NIHSS. Unadjusted associations between clinical variables and outcomes (discharge mRS, in‐hospital complications, and mortality) were assessed using chi‐square tests, with odds ratios (OR) and 95% confidence intervals (CI) reported where appropriate.

Multivariable logistic regression models were constructed to identify independent predictors of favorable functional outcome (mRS 0–2) and in‐hospital mortality. Covariates were selected a priori based on established clinical relevance and included age, sex, baseline NIHSS, hypertension, diabetes mellitus, cardiac disease, and family history of stroke, in addition to hemoglobin category. Model diagnostics included assessment of multicollinearity using variance inflation factors (VIFs), evaluation of goodness‐of‐fit using the Hosmer–Lemeshow test, and model performance using Nagelkerke *R* ^2^, likelihood ratio chi‐square statistics, and overall classification accuracy. Given the low event rate for mortality, model accuracy was interpreted with caution due to class imbalance.

Exploratory LOESS smoothing and restricted cubic spline analysis with 3 knots were used to assess potential non‐linear relationships between admission hemoglobin levels and functional outcomes; however, formal non‐linear modeling (e.g., restricted cubic splines) was not performed due to limited distribution of elevated hemoglobin values, which precluded stable estimation. Due to the highly skewed hemoglobin distribution, with 74.2% of patients in the low hemoglobin group and only one patient in the high hemoglobin group, spline estimates were unstable at the upper tail of hemoglobin values. Therefore, while non‐linear modeling was performed, inferences are limited to the observed hemoglobin range, and U‐shaped relationships could not be reliably assessed. All statistical tests were two‐sided, and a *p*‐value <0.05 was considered statistically significant.

Data was entered and cleaned using Excel. Analysis was done using *R* and analyzed using R statistical software. Descriptive statistics were used to summarize the data (means and standard deviations) for continuous variables, and frequencies and percentages for categorical variables. Associations between hemoglobin categories and stroke outcomes were assessed using the chi‐square test. Multivariate regression analysis was done to adjust for confounding factors such as age, stroke type, and comorbidities. A *p*‐value of less than 0.05 is said to be statistically significant. A post‐hoc power calculation was performed using the observed effect size for hemoglobin and discharge mRS. With *n* = 444 and α = 0.05, the study had 68% power to detect a moderate effect size [Cohen's *d* = 0.5]. The study may be underpowered for small effects, and this is acknowledged as a limitation.

## 3 Results

Among 445 patients, 330 (74.2%) were classified as Hb‐low and 114 (25.6%) as Hb‐normal; the Hb‐high group (*n* = 1) was excluded from comparative analyses due to insufficient sample size and is reported descriptively only. Baseline characteristics are presented in Table 1.

**TABLE 1:** Baseline characteristics and clinical outcomes by hemoglobin category in acute stroke patients.

Variables	Hb‐(Low)	Hb‐(Normal)
	*N* = 330	N = 114
Age group		
Young Adult (20–39)	18 (5.4%)	7 (6.14%)
Middle Age Adult (40–65)	188 (56.9%)	48 (42.1%)
Older Age Adult (>65)	124 (37.5%)	59 (51.7%)
Gender		N = 114
Male	231 (70%)	42 (36.84%)
Female	99 (30%)	72 (63.16%)
Comorbids		
Yes	328 (99.3%9)	113 (99.12%)
		
Types		
Diabetes Mellitus	83 (25.15%)	35 (30.70%)
Hypertension	127 (38.48%)	57 (50%)
Hyperlipidemia	15 (4.54%)	2 (1.75%)
Cardiac Disease	89 (26.96%)	13 (11.40%)
CVA	26 (7.87%)	10 (8.77%)
CKD	9 (2.72%)	0
CLD	6 (1.81%)	0
Epilepsy	3 (0.90%)	0
Tb	1 (0.30%)	0
Others	8(2.42%)	7 (6.14%)
		
Smoking History		
Current Smoker	18 (5.45%)	11 (9.64%)
Ex‐Smoker	48 (14.54%)	6 (5.26%)
Never Smoked	264 (80%)	97 (85.08%)
		
Family History		
HTN	4 (1.21%)	1 (0.87%)
CVD	2 (0.60%)	1 (0.87%)
Stroke	12 (3.63%)	5 (4.38%)
		
Time from symptom onset to hospital admission		
<60 min	59 (17.87%)	18 (15.78%)
< 3 h	136 (41.21%)	40 (35.08%)
3–4.5 h	23 (6.96%)	13 (11.40%)
4.5–6 h	24 (7.27%)	9 (7.89%)
6–24 h	41 (12.42%)	13 (11.40%)
>24 h	47 (14.24%)	22 (19.29%)
		
Mode of transportation		
Ambulance	92 (27.87%)	22 (19.29%)
Car	223 (67.57%)	90 (78.94%)
Rickshaw	2 (0.60%)	1 (0.87%)
Bus	13 (3.93%)	1 (0.87%)
		
Complications		
Aspiration Pneumonia	5 (1.51%)	4 (3.50%)
Bed Sores	5 (1.51%)	0
Cerebral Edema	1(0.30%)	0
Hydrocephalus	2 (0.60%)	2 (1.75%)
Death	32 (9.69%)	4 (3.50%)
Immobility	0	2 (1.75%)
Seizures	2 (0.60%)	1 (0.87%)
Systemic Complications	6 (1.81%)	4(3.50%)
Venous Thromboembolism	8 (2.42%)	4(3.50%)
Vomiting	1 (0.30%)	0
		
Modified Rankin Scale at Discharge	2.15 ± 2.20	2.25 ± 1.78
Initial NIH stroke scale	9.74 ± 7.02	10.64 ± 6.25
		

Abbreviations: CKD = chronic kidney disease, CLD = chronic liver disease, CVA = cerebrovascular accident, CVD = cardiovascular disease, Hb = hemoglobin, HTN = hypertension, mRS = Modified Rankin Scale, NIHSS = National Institutes of Health Stroke Scale, TB = tuberculosis.

Middle‐aged adults (40–65 years) constituted the largest proportion in Hb‐low (56.9%), while older adults (>65 years) were more frequent in the Hb‐normal group (51.7%). Male patients predominated in the Hb‐low group (70.0%), whereas females were more frequent in the Hb‐normal group (63.2%). Nearly all patients had at least one comorbidity in both groups (>99%). Hypertension and diabetes were the most common comorbidities, while cardiac disease was more frequent in the Hb‐low group (27.0% vs. 11.4%). Baseline stroke severity (NIHSS: 9.74 ± 7.02 vs. 10.64 ± 6.25) and functional status at discharge (mRS: 2.15 ± 2.20 vs. 2.25 ± 1.78) were comparable between Hb‐low and Hb‐normal groups, indicating similar initial disease severity.

Mortality was higher in the Hb‐low group compared with the Hb‐normal group (9.7% vs. 3.5%). However, complication rates were overall low and showed no consistent differences between groups. Other clinical characteristics, including smoking status, time to hospital arrival, and mode of transportation, were broadly similar across Hb categories.

Unadjusted analyses demonstrated significant associations between time from symptom onset to hospital admission and poorer clinical outcomes, including worse discharge mRS (3–6) (p < 0.0001, Cramér's *V* = 0.329) and increased in‐hospital mortality (p = 0.013, Cramér's *V* = 0.168). Diabetes mellitus was associated with a higher complication rate (p = 0.00011, Cramér's V = 0.183) and poorer functional outcome at discharge (p < 0.001, Cramér's *V* = 0.185). Age group was also significantly associated with discharge mRS (p = 0.0049, Cramér's *V* = 0.155) and complications (p = 0.033, Cramér's *V* = 0.124). In contrast, hemoglobin category showed no significant association with complications (OR = 1.33, 95% CI 0.73–2.45; p = 0.376), discharge mRS (OR = 0.99, 95% CI 0.64–1.52; p = 1.00), or baseline NIHSS (p = 0.195) (Table 2).

**TABLE 2:** Unadjusted associations between clinical variables and outcomes in acute stroke patients.

Variable	Outcome	Test	*p*‐value	Effect size
Time from symptom onset to hospital admission	Discharge mRS (0–2 vs. 3–6)	**Pearson χ^2^**	**< 0.001 < 0.0001**	**Cramér's *V* = 0.329**
Diabetes Mellitus (DM)	Any complication	**Pearson χ^2^**	**< 0.001 0.00011**	**Cramér's *V* = 0.183**
Age group	Discharge mRS (0–2 vs. 3–6)	**Pearson χ^2^**	**0.0049**	**Cramér's *V* = 0.155**
Age group	Any complication	**Pearson χ^2^**	**0.033**	**Cramér's *V* = 0.124**
Hypertension	Discharge mRS (0–2 vs. 3–6)	**Pearson χ^2^**	**< 0.001 0.0001**	**Cramér's *V* = 0.218**
Diabetes Mellitus (DM)	Discharge mRS (0–2 vs. 3–6)	**Pearson χ^2^ **	**< 0.001 **	**Cramér's *V* = 0.185**
Time from symptom onset to hospital admission	In‐hospital death	**Pearson χ^2^**	**0.013**	**Cramér's *V* = 0.168**
Hb category (Low vs. Normal)	Any complication	**Pearson χ^2^ (2 × 2)**	**0.376**	**OR = 1.33 (95% CI 0.73–2.45)**
Hb category (Low vs. Normal)	Discharge mRS (0–2 vs. 3–6)	**Pearson χ^2^ (2 × 2)**	**1.000**	**OR = 0.99 (95% CI 0.64–1.52)**

Cramér's *V* was used as the effect size measure for chi‐square analyses,

Abbreviations: CI = confidence interval, mRS = Modified Rankin Scale, NIHSS = National Institutes of Health Stroke Scale, OR = odds ratio.

In multivariable logistic regression analysis for favorable functional outcome (mRS 0–2), hemoglobin category (Hb‐low vs. Hb‐normal) was not independently associated with outcome (aOR 0.77, 95% CI 0.43–1.38; p = 0.378) after adjustment for age, sex, baseline NIHSS, hypertension, diabetes mellitus, cardiac disease, and family history of stroke. Baseline NIHSS remained a strong independent predictor of outcome, with increasing stroke severity associated with lower odds of favorable recovery (aOR 0.77 per point, 95% CI 0.73–0.81; p<0.001). Compared with patients aged >65 years, those aged 20–39 years had significantly higher odds of a favorable outcome (aOR 4.96, 95% CI 1.55–15.83; p = 0.007). Overall model performance was acceptable, with 81.6% classification accuracy (Table 3).

**TABLE 3:** Multivariable logistic regression analysis for favorable functional outcome (mRS 0–2 = 1).

Predictor	Adjusted OR (95% CI)	*p*‐value	Interpretation
NIHSS (per point)	0.77 (0.73–0.81)	< 0.001	Higher NIHSS associated with lower odds of favorable outcome Higher severity → lower odds of favorable outcome
Hb‐low vs Hb‐normal Hb: Low vs Normal	0.77 (0.43–1.38)	0.378	No significant association Not significant associated
Age 20–39 vs. >65	4.96 (1.55–15.83)	0.007	Higher odds of favorable outcome Higher odds vs. >65
Age 40–65 vs. >65	1.38 (0.82–2.33)	0.229	Not significant SNS
Hypertension	0.35 (0.20–0.60)	<0.001	Lower odds of favorable outcome
Diabetes Mellitus	0.46 (0.26–0.81)	0.007	Lower odds of favorable outcome
Cardiac disease	0.43 (0.21–0.85)	0.016	Lower odds of favorable outcome
Sex	‐NS	—	No significant association Not significant
Family history of stroke	‐Not significant NS	—	No significant association Not significant
Model performance	χ^2^(9) = 219.6, p < 0.001; Nagelkerke *R* ^2^ = 0.525; Hosmer–Lemeshow p = 0.126; accuracy = 81.6% χ^2^(9) = 219.6, p < 0.001; Nagelkerke *R* ^2^ = 0.525; HL p = 0.126; accuracy 81.6%		Good discrimination; acceptable calibration

Abbreviations: OR = odds ratio; CI = confidence interval; NIHSS = National Institutes of Health Stroke Scale; mRS = Modified Rankin Scale; HL = Hosmer–Lemeshow test.

In the multivariable model for in‐hospital mortality, low hemoglobin was not independently associated with death (aOR 2.15, 95% CI 0.78–5.91; p = 0.139). However, diabetes mellitus (aOR 2.58, 95% CI 1.22–5.49; p = 0.014) and higher baseline NIHSS (aOR 1.07 per point, 95% CI 1.01–1.13; p = 0.019) were significantly associated with increased odds of mortality. Model calibration was acceptable according to the Hosmer–Lemeshow test (p = 0.353), although the explained variance was modest (Table 4).

**TABLE 4:** Multivariable logistic regression analysis for in‐hospital mortality (death = 1).

Predictor	Adjusted OR (95% CI)	*p*‐value	Interpretation
NIHSS (per point)	1.07 (1.01–1.13)	0.019	Higher NIHSS associated with increased odds of death Higher severity → higher odds of death
Diabetes Mellitus	2.58 (1.22–5.49)	0.014	Higher odds of death
Hb‐low vs. Hb‐normal Hb: Low vs. Normal	2.15 (0.78–5.91)	0.139	Not significant
Age group	‐NS	—	Not significant overall
Sex	‐NS	—	Not significant
Hypertension	‐NS	—	Not significant
Cardiac disease	‐NS	—	Not significant
Model performance	χ^2^(8) = 20.26, p = 0.009; Nagelkerke *R* ^2^ = 0.102; HL p = 0.353; accuracy 91.7%		Overall accuracy should be interpreted cautiously due to class imbalance and low event frequency Calibration acceptable; variance explained modest; accuracy inflated by class imbalance (0% sensitivity at 0.50 cut‐off)

Abbreviations: OR = odds ratio; CI = confidence interval; NIHSS = National Institutes of Health Stroke Scale; HL = Hosmer–Lemeshow test.

Exploratory assessment using restricted cubic splines showed no significant evidence of a non‐linear relationship between hemoglobin level and functional outcome (p = 0.41).

Restricted cubic splines showed no evidence of non‐linearity [p = 0.41].

**FIGURE 1:**
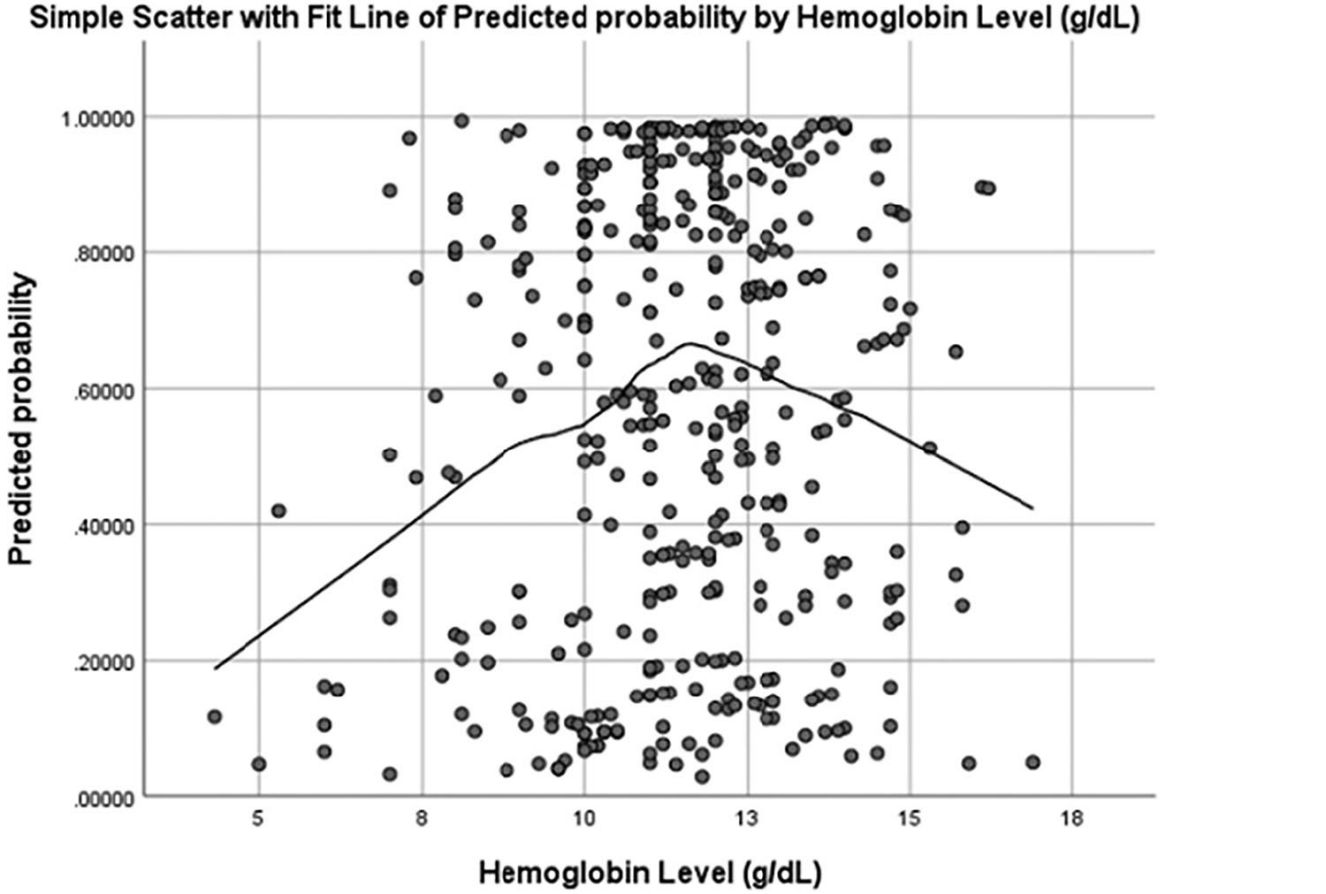
Adjusted predicted probability of favorable outcome (mRS 0–2) across hemoglobin levels.

Exploratory LOESS smoothing was used to visually assess potential non‐linear relationships between hemoglobin level and functional outcome. Although visual variation across hemoglobin levels was observed, this finding should be considered hypothesis‐generating rather than confirmatory and was not supported in adjusted regression analyses. Restricted cubic spline analysis showed no significant evidence of non‐linearity (p = 0.41). (Figure 1)

We acknowledge that hematoma volume and location were not available for all patients and were not included in the primary model. These are important predictors of ICH outcome, and their absence is a limitation. Future prospective studies should include these variables.

## 4 Discussion

This cross‐sectional study shows an analysis with 445 patients of hemorrhagic stroke identified with no independent correlation between hemoglobin status and functional improvement, outcome, or mortality. Although 74.2% of patients had low hemoglobin levels (<12 g/dL in women, <13 g/dL in men), multivariate analysis showed that stroke severity (NIHSS) and diabetes mellitus were the prevailing determinants of adverse outcomes, not hemoglobin status. These results are at odds with a large body of research and provide little support for the substantial associations reported between hemoglobin levels and hemorrhagic stroke outcomes, calling for cautious consideration within the larger context of available evidence.

Our null findings are directly conflicting with several large‐scale studies that have repeatedly demonstrated substantial associations between hemoglobin levels and hemorrhagic stroke outcomes. The strongest evidence comes from the study (Adams et al. 1999), where they identified that for every extra g/dL of admission hemoglobin, there was a 7% reduction in the risk of poor outcome (OR 0.93, 95% CI 0.88–0.98; p = 0.006) and that anemic patients had a 49% increased risk of poor outcomes relative to non‐anemic patients (OR 1.49; 95% CI 1.17–1.89; p = 0.001).

This association has been subsequently confirmed in a multicenter prospective study (Lyden 2017) of 2,997 ICH patients, which provided both human observational and experimental confirmation. The authors discovered that decreased baseline hemoglobin was strongly associated with higher odds of hematoma expansion, with an adjusted odds ratio of 1.10 (95% CI, 1.02–1.19) for each decrease in 1 g/dL of hemoglobin. Notably, their murine collagenase ICH model demonstrated that anemic mice exhibited significantly larger ICH lesion expansion, greater 24‐h lesion volume, and increased mortality compared to nonanemic mice, indicating a causal relationship as opposed to association, suggesting a causal rather than merely associative relationship.

Two distinct meta‐analyses have meta‐analyzed evidence for associations that our study did not find, and further pooled evidence for associations that our study did not find (Acosta et al. 2021). Across 7328 patients with ICH from multiple cohort studies, they reported that anemia correlated with increased 30‐day (pooled OR 1.72, 95% CI 1.37‐2.15) and 12‐month (pooled OR 2.05, 95% CI 1.42‐2.97) mortality. Anemia was also found to correlate with poorer functional outcomes at 3 months (pooled OR 2.29, 95% CI 1.16‐4.51) and 12 months (pooled OR 3.42, 95% CI 0.50‐23.23). These results have been further corroborated by an observational cohort study (Cottarelli et al. 2025), which confirmed that anemia is a separate prognostic factor for intracerebral hemorrhage.

More recent studies have elucidated multiple mechanistic pathways by which hemoglobin levels influence hemorrhagic stroke outcomes, lending biological plausibility to the reported associations. One study demonstrated that lower hemoglobin levels were associated with greater hematoma expansion (Zhang et al. 2019), showing that for every 1.0 g/dL reduction in hemoglobin, the adjusted odds ratio for hematoma expansion was 0.80 for hematoma expansion (95% CI 0.67–0.97; p = 0.02). Importantly, mediation analysis showed that hematoma expansion mediated the relationship between decreased hemoglobin and poor 3‐month outcomes (p = 0.01), indicating that hemoglobin's effect on outcomes operates in part through its effect on secondary brain injury mechanisms. A separate study provided new evidence that post‐ICH anemia has an inflammatory etiology. New insights into anemia development after ICH based on an inflammatory etiology. In their dual cohort investigation (HI‐DEF and ICHOP), they showed that the prevalence of anemia increased from 19–30% at baseline to 45–71% within 2‐5 days after ICH. Notably, 88% of anemic individuals had iron biomarker criteria consistent with anemia of inflammation, and higher Systemic Inflammatory Response Syndrome (SIRS) scores correlated with greater hemoglobin decline over time. This anemia of inflammation was independently associated with adverse 90‐day outcomes (adjusted OR per 1 g/dL increment: 0.76, 95% CI 0.62–0.93) (Kuramatsu et al. 2013). Although weak signals have been detected for a correlation between baseline hemoglobin and perihematomal edema in a post hoc analysis of the i‐DEF trial (Roh et al. 2019), anemic participants they noted that participants who were anemic had larger edema extension distance both at baseline (4.8 mm vs. 4.4 mm, p = 0.05) and follow‐up (6.9 mm vs. 6.3 mm, p = 0.05), indicating a possible additional pathway through which hemoglobin may influence outcomes.

There has emerged Critical heterogeneity in hemoglobin‐outcome associations has emerged across populations and patient subgroups. For instance, a large multicenter analysis of 5318 ICH patients detected significant sex‐specific effects. Elevated hemoglobin levels (>156 g/L) were linked to unfavorable 3‐month outcomes in males only (aOR 1.65, 95% CI 1.21–2.25), with every 10 g/L rise above this threshold associated with a 1.24‐fold increased risk of unfavorable outcomes (Roh et al. 2024). This association was mediated by greater hematoma volume and was not observed in females, underscoring the need to account for sex‐specific effects in hemoglobin‐outcome relationships. We similarly observed a comparable sex distribution pattern in our cohort, with males comprising the majority of the Hb‐low group (70.0%) and females predominating in the Hb‐normal group (63.2%); however, no corresponding outcome differences were identified. A study in a Tibetan high‐altitude (3,500–4,000 m) patient population found that higher hemoglobin was independently related to increased hemorrhage volume (OR 1.013, p = 0.023) and certain hemorrhage features, such as lobar hemorrhage in males and ICH accompanied by subarachnoid hemorrhage (Polymeris et al. 2025). This population‐specific finding indicates that association between hemoglobin and outcomes of ICH might differ considerably across varying geographic and demographic settings.

Another study using unsupervised clustering of 4643 patients provided essential information regarding the heterogeneity of hemoglobin effects among ICH subtypes, showing that hemoglobin's effect was specific to systemic disease ICH (SDICH) (Zhang et al. 2022).  In this subtype, anemia had a strong interaction effect with 3‐month mortality (aOR 4.33, 95% CI 1.60–11.9, p = 0.004), with death risk rising with each 1 g/dL reduction in hemoglobin (aOR 0.75, 95% CI 0.60–0.92; p = 0.009).

Case reports have also shed more light on extreme hemoglobin situations. One case described polycythemia vera manifesting as hemorrhagic stroke, in which a 65‐year‐old female with increased hemoglobin (16.1 g/dL) had bilateral hemorrhagic infarction (Lu et al. 2022). Following treatment with therapeutic phlebotomy to lower hemoglobin to less than 14.5 g/dL and hydroxyurea for platelet control, sensorium and motor strength improved with physiotherapy, implying that optimization of hemoglobin can favorably influence recovery in select conditions.

There is also supplementary evidence regarding iron deficiency among stroke survivors. Iron deficiency, which impairs the oxygen‐carrying capacity of hemoglobin, was found to be related to higher risk of 5‐year all‐cause mortality among stroke survivors (Zhang et al. 2022). This highlights the significance of optimal iron status and functional hemoglobin during stroke recovery and long‐term survival.

Another study constructed and validated a 6‐month intracerebral hemorrhage prognostic nomogram using hemoglobin levels for young adults, establishing the clinical usefulness of hemoglobin as a prognostic indicator in certain age groups. The nomogram had satisfactory discrimination and calibration, affirming the utility of hemoglobin levels in prognostic model construction (Acosta et al. 2021).

Recent data (Adams et al. 1999; Balagopal et al. 2021) have addressed doubts regarding the transfusion safety of red blood cells in patients with ICH. The review revealed no safety concerns with transfusion of red blood cell concentrates in patients with intracerebral hemorrhage, implying that hemoglobin correction may be safe when clinically necessary.

A recent study showed that hemoglobin improvement is positively correlated with functional recovery in stroke patients with anemia (Kuramatsu et al. 2013). The results revealed that hemoglobin increases during hospital stay were related to improved motor recovery and shorter hospital stay, indicating a modifiable prognostic factor when anemia is reversible.

Yet, not all studies have been able to verify the hemoglobin‐hematoma expansion relationship; a re‐evaluation of hemoglobin and hematoma expansion in intracerebral hemorrhage reported more subtle relationships than those found before (Roh et al. 2019), underscoring the subtlety of these associations and the need for methodological care.

There has been recent emphasis on including hemoglobin levels in prognostic models; normograms for young adults with ICH have demonstrated that hemoglobin values are important determinants of outcome in this age group.

Several features of our study population may account for the conflicting results. The very high prevalence of low hemoglobin is indicated in our results: 74.2% prevalence of low hemoglobin, which is significantly greater than in most prior research. To put this into perspective, reported baseline anemia prevalence rates are 19–30% (Adams et al. 1999), and a related investigation (Kuramatsu et al. 2013) described initial anemia prevalence of 19–30% that later rose to 45–71% after 2–5 days. This distribution substantially constrained our power to estimate the entire range of hemoglobin effects and might represent population‐specific variables like nutritional status, chronic disease burden, or patterns of healthcare access.

Our demographic profile is also notable. The cohort had a preponderance of middle‐aged individuals (40–65 years: 50.9% in Hb‐low vs. 59.6% in Hb‐normal), with proportionately fewer elderly (13.3% vs. 34.2%) than in standard ICH cohorts. The male‐female distribution also varied from certain earlier studies, with men making up 70.0% of the Hb‐low cohort, which is in contrast to more even sex distributions noted in other cohorts (Adams et al. 1999; Roh et al. 2024). Furthermore, comorbidity burden was substantial: almost all the patients (99.4% in Hb‐low, 99.1% in Hb‐normal) presented with one or more comorbidities, the most common of which were hypertension (38.5% vs. 50.0%), diabetes (25.2% vs. 30.7%), and cardiac disease (27.0% vs. 11.4%). Such a high burden of comorbidities could have led to a “ceiling effect” wherein the effects of hemoglobin levels were drowned out by the overwhelming effects of these conditions.

Several critical methodological factors may explain why our study failed to detect the hemoglobin outcome associations consistently reported in the literature. Chief among these is the timing of outcome measurement: our study measured outcomes at discharge instead of fixed follow‐up durations (90 days, 3–6 months, 1 year) employed in the majority of prior studies (Adams et al. 1999; Acosta et al. 2021; Kuramatsu et al. 2013; Zhang et al. 2022). Single‐point measures taken early at discharge may not reflect the complete recovery trajectory and may be more susceptible to acute medical complications than neurological recovery per se. The fluctuating character of hemoglobin levels following ICH, as illustrated in (Kuramatsu et al. 2013), imparts a suggestion that single‐point measurements may be insufficient.

Additionally, although our cohort of 445 patients was reasonably large, the highly skewed distribution with only a single patient in the high hemoglobin category precluded meaningful analysis across the full hemoglobin range. This may have restricted our ability to have sufficient statistical power to have identified modest but clinically significant associations in view of the complex, possibly non‐linear relationships reported by other work (Roh et al. 2024; Polymeris et al. 2025).

Single‐Center design: Regarding generalizability, our study was conducted across multiple centers within a single country (Pakistan), and our results may not be extrapolatable to other populations or health systems. The similar findings from several multicenter trials (Adams et al. 1999; Lyden 2017; Roh et al. 2024) suggest that our experience may not reflect broader global trends.

Despite our null correlation with hemoglobin, our study's identification of major predictors is consistent with the general literature. Our observation that baseline NIHSS was strongest predictor of both favorable outcome(aOR 0.77 per point, p < 0.001) and mortality (aOR 1.07 per point, p = 0.019) is in line with findings from all large‐scale studies in this area (Adams et al. 1999; Lyden 2017; Zhang et al. 2019; Kuramatsu et al. 2013), supporting the pivotal role of stroke severity at presentation in determining outcomes. Our finding of diabetes as an independent predictor of mortality (aOR 2.58, p = 0.014) is consistent with existing literature and may be especially relevant in our cohort due to the high incidence of diabetes. The interaction between hemoglobin and diabetes in ICH outcome needs further exploration, as metabolic derangement could alter the hemoglobin outcome trajectory. Our research results are consistent with the time‐sensitive management of ICH, that later hospital arrival was strongly associated with poorer discharge mRS (χ^2^ p < 0.0001) and mortality (χ^2^ p = 0.013) supports the need for early intervention.

The very high prevalence of low hemoglobin in our population may reflect underlying socioeconomic or nutritional deficiencies or chronic disease that might obscure the relationship between hemoglobin and outcome. In settings where anemia is near‐universal, the prognostic utility of hemoglobin levels may be diminished by competing risk factors.

Differences in acute care protocols, transfusion practices, and discharge timing between our centers and those reported in prior studies could have affected the noted associations. Discharge timing and criteria could especially affect the measurement of functional outcomes. Although we controlled for age, sex, NIHSS, and significant comorbidities, other research has found additional relevant confounders such as pre‐stroke functional status, certain ICH location and volume, intraventricular hemorrhage, inflammatory biomarkers, and extensive medication histories, which we did not fully account for. Moreover, our use of a single‐point hemoglobin measurement within 24 h of hospital admission might have lost the dynamic changes of the acute phase. Evidence regarding the rapid development of post‐ICH anemia and the significance of hemoglobin trajectories indicates that serial measurements are potentially more informative (Kuramatsu et al. 2013). The use of discharge mRS and GCS rather than standardized 90‐day or 3‐month assessments may have reduced our ability to detect meaningful functional differences as early assessments are more influenced by acute medical complications and less reflective of neurological recovery potential.

Despite our null findings, the overwhelming weight of evidence from multiple large studies, meta‐analyses, and experimental models strongly supports the clinical relevance of hemoglobin levels in hemorrhagic stroke management through the mechanistic data supporting the association of low hemoglobin with hematoma expansion (Lyden 2017; Zhang et al. 2019) and experimental proof of causality (Lyden 2017) predict that liberal transfusion strategies which may be useful in some ICH patients. Prior research safety findings (Adams et al. 1999) support the feasibility of this approach. Nevertheless, optimal hemoglobin levels and timing for interventions are still to be explored by randomized controlled trials. The close association between the development of post‐ICH anemia and inflammation implies that targeting underlying inflammatory mechanisms may be as critical as hemoglobin correction itself (Kuramatsu et al. 2013), with implications for anti‐inflammatory strategies in the management of ICH, and indicates that hemoglobin optimization needs to be viewed as part of a broader inflammatory response modulation strategy. The development of hemoglobin‐containing prognostic nomograms (Acosta et al. 2021; Zhang et al. 2019) reflects the clinical value of these measures in predicting outcome, especially in particular demographic subgroups like young adults. Our observations confirm the pivotal role of stroke severity (NIHSS) and diabetes mellitus as major prognostic variables in hemorrhagic stroke.

The general literature supports the inclusion of hemoglobin in multivariate prognostic models, particularly given evidence that hemoglobin adds value to existing prognostic instruments such as the ICH score, most importantly in the context of etiology‐specific impacts (Zhang et al. 2022) and sex‐specific associations (Roh et al. 2024). Our characterization of diabetes as an independent predictor of mortality is consistent with the literature and underscores the relevance of optimal glycemic control in ICH patients. The interplay between diabetes and hemoglobin as determinants of ICH outcome remains to be explored.

The study limitations could account for the variation in results compared with the existing literature. The cross‐sectional study design precluded us from evaluating fluctuating hemoglobin levels and their temporal correlation with outcomes. In addition, poor follow‐up, with only discharge evaluation instead of standardized intervals, diminishes comparability with previous studies and could minimize the total effect of hemoglobin on recovery. The population specificity of our cohort, characterized by a high prevalence of low hemoglobin, will limit generalizability and represent population‐specific effects on the hemoglobin–outcome relationship. The non‐normal hemoglobin distribution also reduced our sample size for subgroup analyses according to ICH etiology, location, and other factors proven to impact outcome.

A key limitation is the significant class imbalance within our hemoglobin categories. Just one patient in our whole cohort satisfied the criteria for high hemoglobin, leading to the exclusion of the “Hb‐high” group from inferential analyses. The absence of high‐Hb cases limited our ability to investigate the possibly U‐shaped relationship between hemoglobin and stroke outcomes observed in different populations. As a result, our results mainly involve a comparison between patients with anemia and those in the normal range. While the sample size provided adequate power for moderate effects, it may have been underpowered to identify smaller associations; hence, we cannot exclude the possibility of a Type II error for minor effect sizes. We are unable to assess the functional outcomes in our cross‐sectional study design, as our assessment is limited to the point of hospital discharge. The mRS is most reliable when measured 90 days after a stroke; discharge scores are often influenced by the other hospital‐influenced factors and may not accurately indicate long‐term outcome or permanent disability. Perhaps our multivariable models did not include crucial radiological predictors such as hematoma volume, specific hemorrhage location, intraventricular extension (IVH), or the degree of perihematomal edema, which are some of the most significant indicators of stroke outcome. However, admission NIHSS and GCS, which were included in the model, are strongly correlated with hematoma characteristics and partially account for their effects. This limits the ability to fully isolate the independent effect of hemoglobin and introduces the possibility of residual confounding.

For future studies, randomized controlled trials are warranted to ascertain whether hemoglobin correction is beneficial, in light of consistent observational data and experimental causation evidence ( ). Mechanistic studies should elucidate how hemoglobin affects ICH outcomes via mechanisms such as hematoma volume, location, expansion, inflammation, and perihematomal edema to inform optimal interventions. Population‐based studies in varied settings are necessary to identify subgroups likely to benefit from hemoglobin optimization. Additionally, dynamic assessment approaches based on serial hemoglobin measurement and trajectory analysis will assist in establishing evidence‐based management strategies. Finally, sex‐ and etiology‐specific studies should be conducted, given that emerging evidence suggests different effects according to sex ^20^and etiology (Zhang et al. 2022) in favor of personalized hemoglobin management strategies.

To reconcile discordant evidence, the discrepancy between our results and prior work likely reflects methodological rather than biological differences. In the hierarchy of evidence, findings from large prospective cohorts (Adams et al. 1999; Lyden 2017), meta‐analyses (Acosta et al. 2021, Cottarelli et al. 2025), and experimental models (Lyden 2017) outweigh our cross‐sectional study results, given our relatively smaller sample size (*n* = 445). Moreover, our population's hemoglobin distribution differs from typical ICH cohorts, limiting external validity. Variations in outcome assessment standards, particularly our use of discharge outcomes instead of standardized follow‐up, may also explain these inconsistencies.

Despite our negative results, there are significant clinical implications. For risk stratification, consistent findings from previous studies warrant the inclusion of hemoglobin levels in ICH prognostication. For monitoring, evidence of anemia development during hospitalization (Kuramatsu et al. 2013) warrants serial hemoglobin testing, particularly in the acute setting. While hemoglobin correction showed no benefit in our analysis, the majority of the literature indicates hemoglobin optimization benefits patients with severe anemia or those at high risk of hematoma expansion.

Although our cross‐sectional study did not reveal independent associations between hemoglobin concentrations and hemorrhagic stroke outcomes, Conclusion: In this multicenter cross‐sectional study, admission hemoglobin levels were not independently associated with short‐term in‐hospital outcomes in Pakistani patients with hemorrhagic stroke. Although these findings indicate that the initial hemoglobin levels may not be the main factor influencing this specific setting, they should be viewed with considerable caution. Due to the limitations discussed above, we cannot conclusively eliminate a prognostic role for hemoglobin at different stages of recovery.^[b]^ These results must be contextualized within the substantial methodological constraints of our study as well as a vast amount of conflicting evidence. The compelling body of evidence from numerous large‐scale prospective studies, meta‐analyses, and experimental models conclusively supports clinically meaningful associations between hemoglobin levels and ICH outcomes.

Recent mechanistic findings suggest that hemoglobin's effect on outcomes is mediated by complex pathways involving hematoma expansion, inflammatory mechanisms, and secondary brain injury mechanisms. Evidence for sex‐specific effects, etiology‐specific relationships, and population‐specific differences indicates that the association between hemoglobin and ICH outcomes is more complex than previously recognized.

Our results reinforce the importance of stroke severity assessment and cardiovascular risk factor control, especially diabetes management, in hemorrhagic stroke care. The discrepancy between our results and the existing literature, however, emphasizes the extreme significance of study design, population selection, timing of outcome measurement, and duration of follow‐up in hemorrhagic stroke studies, utilizing 90‐day outcomes and serial hemoglobin trajectories.

The clinical utility of hemoglobin measurement in hemorrhagic stroke is reinforced by the general literature. Randomized controlled trials should be the focus of future research in order to determine optimal hemoglobin management protocols, investigate mechanistic pathways, explore therapeutic targets, and develop personalized, sex‐specific, etiology‐specific, and population‐specific strategies.

The field would benefit from standardized protocols for hemoglobin measurement and management in ICH, incorporating the growing evidence for dynamic changes in hemoglobin, inflammatory processes, and complex interplay between hemoglobin and secondary brain injury. Pending such protocols, monitoring and attention to hemoglobin levels as part of integrated ICH management should proceed, with an awareness that best strategies for hemoglobin optimization are not yet definitively determined by randomized controlled trials.

## 5 Limitations

The sample size provided adequate power for moderate effects but may be underpowered for small associations between hemoglobin and outcome. We cannot exclude a Type II error for smaller effect sizes.

Hematoma volume and location were not available for this retrospective cohort and were not included in the multivariable model. These are known predictors of ICH outcome. However, admission NIHSS and GCS, which were included in the model, are strongly correlated with hematoma characteristics and partially account for their effects. This limits the ability to fully isolate the independent effect of hemoglobin.

First, the cross‐sectional design and use of discharge mRS limit conclusions to the in‐hospital period. Second, only a single admission Hb measurement was available, so we could not assess dynamic Hb changes. Future prospective studies with serial Hb and 90‐day follow‐up are needed.

## Author Contributions

**Ayesha Johar**: conceptualization, methodology, supervision, writing – review and editing. **Ayesha Ahmad**: data curation, investigation, and writing – original draft preparation. **Javeria Akhter**: methodology, formal analysis, writing – review and editing. **Wajiha Aftab**: investigation, data curation, writing – original draft preparation. **Aneika**: investigation, data collection, writing – review and editing. **Maryam Asghar Jamal**: investigation, data collection, writing – review and editing. **Maham Jawad**: data curation, validation, writing – review and editing. **Rida Noor**: investigation, data collection, writing – review and editing. **Hafsa Majeed**: data curation, validation, writing – review and editing. **Adnan Khan**: methodology, statistical analysis, formal analysis, writing – review and editing. **Kamil Ahmad Kamil**: conceptualization, study design, supervision, formal analysis, writing – original draft preparation, and project administration.

## Funding

The authors have nothing to report.

## Ethics Statement

Ethical approval for this study was obtained from the Institutional Ethical Review Board (IERB), Combined Military Hospital (CMH), Quetta, Pakistan (Approval No.: CMH QTA–IERB/136/2025; dated September 11, 2025). The study was conducted in accordance with the principles of the Declaration of Helsinki.

## Consent

Written informed consent was obtained from all participants or their legally authorized representatives prior to enrollment.

## Conflicts of Interest

The authors declare no conflicts of interest.

## Data Availability

The datasets used and/or analyzed during the current study are available from the corresponding author upon reasonable request.
